# Changes in liver mitochondrial plasticity induced by brain tumor

**DOI:** 10.1186/1471-2407-6-234

**Published:** 2006-10-03

**Authors:** Daniel Pouliquen, Christophe Olivier, Emilie Debien, Khaled Meflah, François M Vallette, Jean Menanteau

**Affiliations:** 1 Inserm, U601, Equipe « Apoptose et progression tumorale », F-44000, Nantes, France; 2 Université de Nantes, Faculté de Médecine, Département de recherche en cancérologie, IFR26, F-44000, Nantes, France; 3 Université de Nantes, Faculté de Pharmacie, F-44000, Nantes, France

## Abstract

**Background:**

Accumulating data suggest that liver is a major target organ of systemic effects observed in the presence of a cancer. In this study, we investigated the consequences of the presence of chemically induced brain tumors in rats on biophysical parameters accounting for the dynamics of water in liver mitochondria.

**Methods:**

Tumors of the central nervous system were induced by intraveinous administration of ethylnitrosourea (ENU) to pregnant females on the 19th day of gestation. The mitochondrial crude fraction was isolated from the liver of each animal and the dynamic parameters of total water and its macromolecule-associated fraction (structured water, H_2_Ost) were calculated from Nuclear Magnetic Resonance (NMR) measurements.

**Results:**

The presence of a malignant brain tumor induced a loss of water structural order that implicated changes in the physical properties of the hydration shells of liver mitochondria macromolecules. This feature was linked to an increase in the membrane cholesterol content, a way to limit water penetration into the bilayer and then to reduce membrane permeability. As expected, these alterations in mitochondrial plasticity affected ionic exchanges and led to abnormal features of mitochondrial biogenesis and caspase activation.

**Conclusion:**

This study enlightens the sensitivity of the structured water phase in the liver mitochondria machinery to external conditions such as tumor development at a distant site. The profound metabolic and functional changes led to abnormal features of ion transport, mitochondrial biogenesis and caspase activation.

## Background

Accumulating data suggest that liver is a major target organ of systemic effects observed in the presence of a cancer. Global analysis such as SELDI-TOF mass spectrometry showed that most if not all identified proteins thus far represent acute-phase reactants produced by the liver in response to inflammation [1]. Pro-inflammatory processes are clearly implicated in the hypermetabolism and weight loss associated with cancer related cachexia [2]. The liver receives metabolites transported by the blood from other tissues, extracts a significant portion of nutrients to provide energy for macromolecular syntheses and export materials. All these processes are potentially affected by the metabolic changes linked to tumor growth. In addition, there are marked alterations in carbohydrate metabolism in the liver which arise from the utilization of glucose by the tumor as the primary energy source [3]. At the subcellular level, mitochondria represent the most important target of cancer systemic effect, firstly through their central role in providing energy, oligoelement metabolism and control of oxidative stress, and secondly by their plasticity in response to metabolic parameters. We previously showed that important modifications of the dynamics of macromolecule-associated water (structured water) occured in the liver tissue, especially in the earliest stage of the development of a chemical-induced lymphoma [4]. However, no evidence has been given so far for a connection between the systemic effect of the presence of a tumor and changes in the dynamics of structured water in liver mitochondria, although this was hypothetized in our previous work [4].

In mitochondria the presence of two membranes and extensive macromolecular crowding confers to interactions between water, macromolecules and membranes a central role in defining efficiency of mitochondrial metabolic processes. The properties of water molecules in the hydration shell of membranes largely depend of the type of lipid headgroup and of the presence of one (MUFA) or multiple (PUFA) double bonds [5]. Both the cholesterol/phospholipid molecular ratio and the (un)saturation of fatty acyl groups contribute to the membrane fluidity, which is an essential parameter for mitochondrial transport of active molecules such as GSH [6]. As a consequence, both the dynamics of water and lipid composition would contribute to the properties of mitochondrial membranes, such as molecular transport and mitochondrial permeability transition, which plays a key role in cell death [7,8].

In this study, we investigated the consequences of the presence of a tumor on the biophysical parameters accounting for the dynamics of water in liver mitochondria, and compared these fluctuations to those induced by short-term fasting, a non-pathological modulator of cellular metabolism. Glioma is an adequate model for this study since this type of tumor is confined to brain and thus systemic effects could be due to the tumor itself and not to the presence of metastases [9]. The characterization of mitochondrial structured water was achieved by Nuclear Magnetic Resonance (NMR) techniques, a powerful and non-destructive method which uses the water molecule as a sensor of the physicochemical properties inside the cell [10]. We have previously shown that NMR investigations on isolated liver mitochondria provide a new insight in the mechanisms of mitochondrial membrane permeabilization and plasticity [7].

We also examined if these parameters were linked to the cholesterol and PUFA content of liver mitochondria, as two keys factors of membrane fluidity and permeability. Finally, because these membrane properties would have an impact on ionic fluxes and PTP we measured the mitochondrial content of ions of high importance in mitochondrial functions and DEVDase activity in the tissue to quantify caspase activation.

## Methods

### Test animals and isolation of liver mitochondria

The experiments reported here comply with the guidelines of the European Union for care and use of animals in research protocols. The animals were housed in polycarbonates rat bredding cages and were given free access to tap water and a standard diet (RM1, Special Diets Services, Witham, Essex, UK). Animals were anaesthetized by ketamine and Rompun^®^, then exsanguinated by decapitation. The mitochondrial crude fraction was isolated from the liver of each animal according to the procedure of Rickwood [11], which allows the whole population of liver mitochondria to be weighed without loss, and the mitochondrial pellet then kept in liquid nitrogen until the moment of NMR measurements as previously described [7]. For the analysis of the influence of fasting, liver mitochondria were isolated from male rats of 6 months of age either normally fed with RM1 or previously fasted for 18 hours.

Tumors of the central nervous system (CNS) were induced according to the procedure of Koestner et al. [12]. At twelve weeks of age four female Ico: OFA-SD (IOPS Caw) rats, purchased from Charles River Laboratories, L'Arbresle, France, were paired with four males of the same strain. The day on which the presence of a vaginal plug was confirmed was defined as day 0 of gestation. On the 19th day of gestation the females received an intraveinous administration of 50 mg/kg of ethylnitrosourea (ENU) (Isopac^®^, Sigma, St Louis, Mi, USA) dissolved in saline. Offsprings were weaned on day 21 after birth, individually marked on the tail and weighed once a week from 17 weeks of age until the moment of necropsy.

### NMR parameters of mitochondrial water dynamics

Spin-lattice (*T1*) and spin-spin (*T2*) relaxation times of the mitochondrial pellets disposed into Pyrex 10 × 75 heavy wall test tubes (Bibby sterilin Ltd, Stone, Stafforshire, England) were measured on a NMS 120 minispec^® ^(Bruker, Wissembourg, France) operating at a Larmor frequency of 20 MHz, as previously described [7]. Thermoregulation was monitored with a Bruker B-VT 2000 variable temperature unit connected to the spectrometer, using nitrogen gas from liquid nitrogen. The sample temperature inside the magnet was determined using a 0.5 mm diameter Inconel K thermocouple placed in the middle of the sample, connected to an SA 32 electronic central unit (AOIP, France). Relaxation times were measured firstly in the frozen state (structured water fraction, *T1sw *and *T2sw*) from 258 K to 237 K, on samples removed from liquid nitrogen, and then at 277 K (total water, *T1obs *and *T2obs*). From the temperature dependency of *T1sw *and *T2sw*, the correlation times for rotational and translational motions, ***τ***_*R *_and ***τ***_*D*_, and their respective enthalpies of activation *E*_*R *_and *E*_*D *_were calculated based on the Arrhenius relation and equations of the model of cross relaxation between macromolecule protons and structured water protons, as previously described by Gallier et al. [13].

### Analysis of cholesterol, PUFA content and ion concentration in mitochondria pellets

Extraction of membrane lipids was made according to the Bligh and Dyer method [14], The PUFA content was determined by ^1^H-NMR spectroscopy analysis of the chloroformic phases. ^1^H-NMR spectra were recorded at 500.13 MHz (Bruker Avance DRX 500) using the ratio of the integrative intensities of methylene protons at 2.8 ppm (-CH=CH-C**H**_2_-CH=CH-) to that of the sum of methylene protons at 1.2 ppm (∑(C**H**_2_)n)(data not shown).

The pellet of precipitated macromolecules together with the hydro-methanolic phase was dried in an oven and then the dried residue was mineralized in HNO_3 _(69%, d = 1.42), heated at 120°C, and then dissolved in bidistilled water (1/3, v/v) and filtrated. The yellowish solution obtained was further analyzed by atomic absorption spectrometry for the determination of the concentrations of Cu^2+^, Mn^2+^, Zn^2+ ^and K^+^. The cholesterol content of liver mitochondria was determined using the chloroformic phases according to the method of Zlatkis and Zak [15].

### Caspase activity assay

Liver tissues were homogenized in lysis buffer supplied by the vendor. The samples were then centrifuged for 30 min at 13,000 rpm at 2°C. The supernatants were removed to new tubes. Protein concentration of cell lysates was determined by using the Bio Rad protein assay kit (Bio Rad Laboratories, Richmond, CA, USA). Caspase activity was monitored by measured the degradation of the fluorometric substrate Ac-DEVD-AMC as previously described [16], using the Assay kit (Promega Corporation, Madison, WI, USA). The final expression of the results was presented as arbitrary units of caspase activity per microgram of protein.

### Statistical analyses

Results were expressed as mean ± SE. The difference between two mean values was analyzed by Student's *t*-test and was considered to be statistically significant when *P *< 0.05.

## Results

### 1) Tumor development induces specific modifications of the structured water phase in liver mitochondria which affect both relaxation times

Below the freezing transition of bulk water to ice, a significant proportion of water in liver mitochondria does not freeze. This water phase called structured water (term now preferred to "bound water", generally used for "dry" powders) corresponds to macromolecule-associated water [17]. The relaxation times of this particular water phase are called *T1sw *and *T2sw*, while those of total water (measured at 277 K) are defined as *T1obs *and *T2obs*.

**Figure 1:**
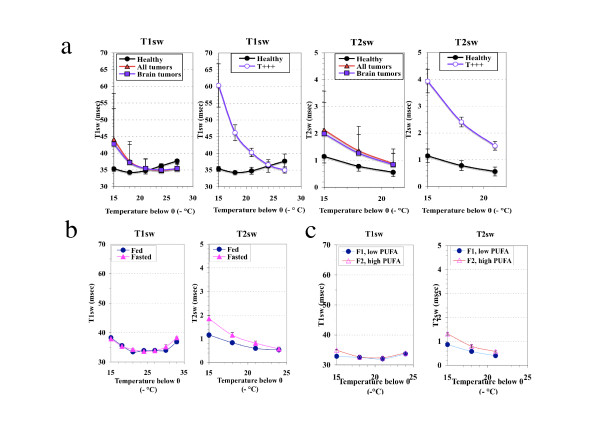
**NMR relaxation times of the structured water phase in the mitochondrial crude fraction of the liver**. **a: **Perturbations induced by brain tumor development (males), **b: **Normal liver: influence of 18 hrs fasting, **c: **Normal liver: changes related to the mitochondrial membranes PUFA content in fed rats, v = 20 MHz.

In order to induce CNS tumor development and to explore systemic effects, an animal model was developed as described in material and methods. Among the group of male rats induced with ENU (n = 32), 68.7% (n = 22) presented a gross tumor, of which 46.9% (n = 15) were located in the brain. Other tumor locations included the spinal cord, connective tissue, bone and prostate. Brain tumors were collected from the 4^th ^to 9^th ^months of age, at different moment of tumor development but mostly in the middle and late phase which was associated with significant body weight loss (mean weight of the brain tumors: 360.2 ± 215.9 mg). Analysis of water dynamics in the liver mitochondria crude fraction from brain tumor-bearing rats (n = 13) compared with that on rats with neither visible signs of pathology nor weight loss (healthy, n = 5) revealed a significant acceleration of the dynamics of H_2_Ost at 258K and 255K (Fig. 1a). In contrast, the changes affecting total water were neither significant for *T1obs *(436.1 vs 394.0 msec) nor for *T2obs *(77.8 vs 74.1 msec) (Table 1). In addition, the NMR data were similar for the all the rats bearing a tumor (all locations) and rats bearing a brain tumor (Fig. 1a). According to the amplitude of change in H_2_Ost dynamics, the brain tumor-bearing rats were easily divided in two sub-groups (bi-modal distribution), the first one being high-sized (n = 10) and moderately different from healthy animals (T), and the second one low-sized (n = 3) and highly different from the healthy rats (T +++). The T+++ subgroup consisted of rats sacrificed at various ages (173, 201 and 244 days) and bearing brain tumors differing in size (326, 824 and 153 mg, respectively). The NMR pronounced acceleration of the physical parameters of H_2_Ost dynamics in the « T +++ » subgroup of brain tumor-bearing rats, compared with healthy rats, is illustrated in Fig 1a.

The brain tumor-bearing rats exhibited a significant decrease in the liver to body weight ratio, particularly in males (all brain tumors vs healthy: 0.0236 ± 0.0044 vs 0.0294 ± 0.0017 for males, 0.0245 ± 0.0031 vs 0.0268 ± 0.0017for females,). To assess if the changes we observed in the parameters of water dynamics were linked to this context, we used short term fasting known to rapidly decrease the liver mass [18]. Fasted rats of 6 months of age presented a 26% increase in *T1obs *(364.2 vs 290.0 msec) and 55% increase in *T2obs *values (88.5 vs 57.1 msec) compared with their values in normally fed rats (Table 1). In the fasted rats the *T2sw *was increased but the *T1sw *was unchanged (Fig. 1b). As a whole, the changes in water observed in the liver mitochondria of the brain tumorbearing rats cannot be attributed to the decrease in liver weight.

We further examined if these data could be related to the membrane PUFA content and to investigate this point we compared rats with low and high levels of PUFA (Fig. 1c). The sample with a low proportion of polyunsaturated fatty acids presented significantly lowered *T1obs *and *T2obs *values as compared with the sample with the highest PUFA content (-10%, *P *< 0.001; and -11%, *P *< 0.001, respectively). As concerns the H_2_Ost, significantly lowered values of the spin-spin relaxation time (*T2sw*) were also observed in the whole -15°C to -21°C temperature range (Fig. 1c), while significant differences between *T1sw *values were restricted to the highest temperature (-15°C)(Fig. 1c). However, the enhancement in *T2sw *increase observed in fasted rats cannot be explained by an even more increase in PUFA content, the fasted to fed rats ratio being 1.7 (0.105 vs 0.062) while that of the high to low PUFA content in normally fed rats was 3.6 (0.126 vs 0.035). Other modifications of the phospholipids composition of the membranes could account for this difference (data not shown). However, with the increase in the level of unsaturation, it is likely that the rate of exchange of bulk water between the intramitochondrial space and the external medium be high enough to produce a significant elevation of *T2obs *and *T1obs *(Table 1). In contrast, the changes in PUFA content cannot account for the dramatic acceleration of H_2_Ost dynamics observed in T+++ brain tumor-bearing rats, as the ratio in this subgroup was decreased by 7.0, compared with healthy rats (0.0077 vs 0.0537).

### 2) Acceleration of H_2_Ost dynamics of liver mitochondria was associated with modifications of cholesterol level and oligoelements status in the brain tumor-bearing rats

Because cholesterol is also a key parameter of the membrane fluidity, and can contribute to limit water permeability through the membrane, we analysed its level in the isolated mitochondria. The cholesterol content of liver mitochondria was significantly higher in the tumor-bearing rats compared with their healthy counterpart (8.0 ± 2.6 μg/mg mitochondrial protein vs 4.9 ± 2.3 μg/mg mitochondrial protein, 0.01 <*P *< 0.02).

**Figure 2:**
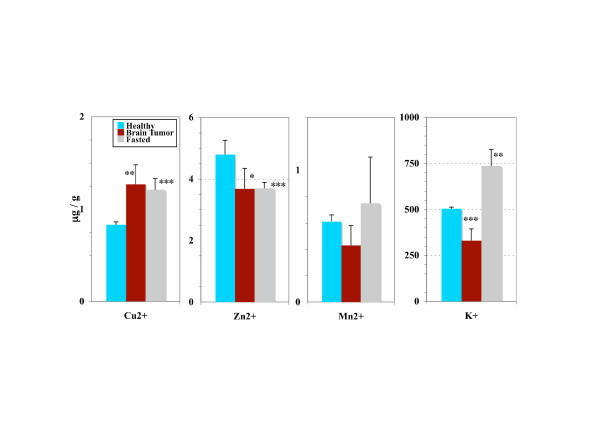
**Ion concentrations in the mitochondrial crude fraction of the liver**. Comparison between brain tumor-bearing rats exhibiting the most pronounced acceleration of the dynamics of the structured water phase (T+++ subgroup), fasted rats and healthy rats (males). Statistical significance (Student's *t*-test) between groups means: 0.02 <P* < 0.05, 0.01 <P** < 0.02, 0.001 <P*** < 0.01.

As perturbations in the membrane plasticity and permeability could induce changes in the ionic fluxes we measured the mitochondrial concentration of ions implicated in the functionnality of this organelle. A very significant decrease in the Zn^2+ ^(3.96 vs 6.84 μg/g, *P *< 0.001) and Mn^2+ ^(0.5 vs 0.77 μg/g, 0.001 <*P *< 0.01) concentrations were observed in brain tumors bearing rats compared with healthy rats, while that in Cu^2+ ^was significantly increased (1.24 vs 1.01 μg/g, 0.02 <*P *< 0.05). The concentration in K^+ ^was also slightly reduced (411.6 vs 525.6 μg/g, 0.02 <*P *< 0.05). Comparison of liver mitochondria ion concentrations between the healthy rats and the subgroup of brain tumor-bearing rats exhibiting the most pronounced acceleration of H_2_Ost dynamics (T+++) showed an elevated copper content, a decrease in Zn^2+ ^and a pronounced decrease in K^+ ^(Fig. 2).

This decrease in ion concentrations (Zn^2+^, Mn^2+ ^and K^+^) that we observed was specific to the tumor bearing rats. Changes in the ion concentrations under fasting were characterized by a very different pattern (drop in Zn^2+ ^but elevation in Cu^2+ ^and K^+^)(Fig. 2).

### 3) The dynamics of the structured water phase in liver mitochondria was associated with an increase in the mitochondrial fraction of liver tissue

**Figure 3:**
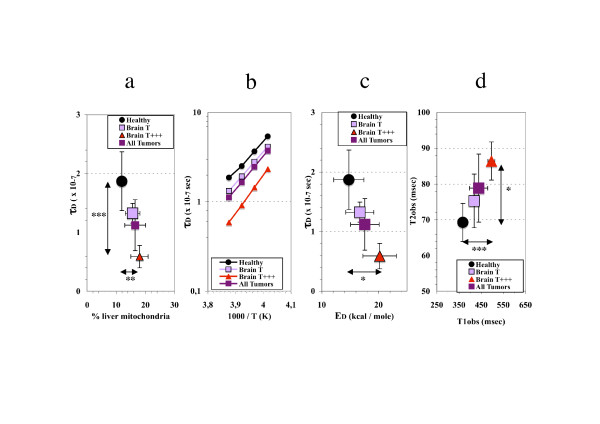
**In brain tumor-bearing rats the structured water dynamics is associated with mitochondrial biogenesis**. Specificities of liver mitochondria from brain tumor-bearing rats and discrimination between healthy rats and subgroups (males): T (moderate increase in structured water dynamics) and T+++ (most pronounced acceleration of structured water dynamics). **a) **Relationship between the weight percentage of mitochondria in the liver and the value of the correlation time for the translational motion of structured water, ***τ***_*D*_, measured at 258K. **b) **Temperature dependency of ***τ***_*D *_in liver mitochondria isolated from healthy and tumor- bearing rats. **c) **Relationship between the correlation time for the translational motion of structured water, ***τ***_*D*_, measured at 258K and its enthalpy of activation, *E*_*D*_. **d) **Elevation in *T1obs *and *T2obs *(relaxation times of total water, measured at 277 K) induced by tumor development. Statistical significance (Student's *t*-test) between groups means; 0.02 <P* < 0.05, 0.01 <P** < 0.02, 0.001 <P*** < 0.01.

We investigated if the changes in water dynamics of liver mitochondria could be related to mitochondrial biogenesis. Therefore we examined the relation between the previously observed most discriminant parameter of mitochondrial water dynamics, ***τ***_*D*_, and the proportion of mitochondria in the different subgroups of rats induced with ENU. A significant rise was observed in the ratio of the wet weight of the mitochondria pellet to the liver weight, in brain tumor-bearing rats compared with healthy rats (0.184 ± 0.060 vs 0.139 ± 0.028, 0.01 <*P *< 0.02). The more ***τ***_*D *_was reduced, the more the proportion of mitochondria was elevated (Fig. 3a). The decrease in ***τ***_*D *_values was accompanied by an elevation of *E*_*D *_(calculated from the slope of the temperature dependency of *T2sw *[7,13] (Fig. 3b)). The more ***τ***_*D *_was reduced, the more *E*_*D *_was elevated (Fig. 3c). The pronounced decrease in ***τ***_*D*_, increase in *E*_*D *_and elevation of the proportion of mitochondria in the liver was also associated with a significant rise in both relaxation times of total water, an observation specific to the T+++ subgroup (Fig. 3d).

### 4) Caspase activity was increased in brain tumor-bearing rats

**Figure 4:**
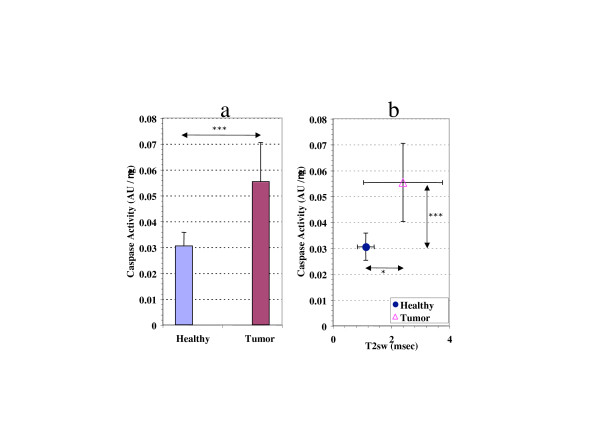
**Influence of brain tumor on the caspase activity in the liver tissue**. **a: **Comparison in caspase activity between brain tumor-bearing rats and healthy rats (males). **b: **Relationship between caspase activity and the increase in *T2sw*. Statistical significance (Student's *t*-test) between groups means, 0.02 <P* < 0.05, 0.001 <P*** < 0.01.

The caspase activity in the liver tissue from brain tumor-bearing rats was significantly higher than in their healthy counterpart (0.001 <*P *< 0.01)(Fig. 4a). As we previously showed that the lower degree of structuration of H_2_Ost observed in apoptotic liver mitochondria was associated to a decrease in ***τ***_*D *_(calculated from the increase in *T2sw*) [7,13], we investigated the relationship between caspase activity and this parameter. The increase in caspase activity was correlated with a significant increase of the *T2sw *(0.02 <*P *< 0.05) when compared with these values in healthy rats (Fig. 4b).

## Discussion

A recent survey of the litterature provided some evidence that cell hydration is the primary factor in the mechanism of carcinogenesis [19]. In this study we investigated the changes induced by brain tumors in water parameters of liver mitochondria. The changes in the water parameters of liver mitochondria that we evidenced were compared with those arising upon transient metabolic disturbance induced by short-term fasting. Finally, the characteristics of water dynamics in the liver mitochondria of tumor-bearing rats were examined in conjunction with the degree of fatty acid unsaturation in the membranes and the levels of cholesterol as other key factors of membrane fluidity. As a whole, the presence of brain tumors affected the ion content of liver mitochondria and enhanced caspase activity in the liver tissue.

Among the different water phases of liver mitochondria, the physical properties of structured water (macromolecule-associated water) were particularly affected by the presence of a brain tumor. According to the amplitude of changes in H_2_Ost dynamics, and whether both types of relaxation times are concerned or not lead to distinguish two separate situations: 1- The first situation, specifically observed in the liver mitochondria of brain tumor-bearing rats was characterized by an acceleration of H_2_Ost dynamics, which was particularly dramatic in the T+++ subgroup (91% and 275% increase in *T1sw *and *T2sw*, respectively) while the increase in *T1obs *and *T2obs *was limited to 36% and 29% of the values measured in healthy rats. These changes mean an important decrease in the correlation times of H_2_Ost for both types of molecular motion together with an increase in the value of their respective enthalpies of activation.

2- The second situation was observed when the lipid composition of the membranes (PUFA) was considered. In that case, ion concentration evolved in non-coherent ways or in low ranges. In that case the acceleration of total water dynamics was even more slight (11% and 12% increase in *T1obs *and *T2obs*, respectively) and the changes affecting H_2_Ost were restricted to *T2sw *in the absence of significant elevation in the enthalpy for this kind of molecular motion. The specific differences observed in *T2sw *in a large temperature range mean that for a given temperature, increasing the degree of lipid membrane unsaturation results in a decrease in the correlation time for translational motion (***τ***_*D*_). Early reports have already shown that a significant proportion of H_2_Ost is associated with phospholipids and that the number of water molecules in the hydration shell depends on several factors including the presence of *cis*-double bonds [20].

The changes we are depicting in brain tumor-bearing rats could not be attributed to fasting since the amplitude of the modifications of H_2_Ost dynamics widely exceeded that observed in fasted rats. In addition, the acceleration of H_2_Ost dynamics in fasted rats was consistent with an elevation of the degree of membrane fatty acid unsaturation while brain tumor-bearing rats exhibited a pronounced decrease in their PUFA content.

Taken as a whole, these results lead to the conclusion that the presence of a malignant tumor in brain induces a loss of the structural order which would have consequences in the physical properties of the hydration shells of liver mitochondria macromolecules.

Changes in the proportion of mitochondrial membrane cholesterol content occured in parallel with changes affecting water dynamics. Given the influence of cholesterol on the dynamics of water in biomembranes, the significant rise in its content observed in the membranes of liver mitochondria of brain tumor-bearing rats suggests that it represents a way to limit water penetration into the bilayer [21], and then to reduce membrane permeability. This feature could also contribute to the absence of significant increase in *T2obs *of mitochondria between the whole population of brain tumor-bearing rats and healthy rats. Although this phenomenon has already been described for hepatoma mitochondria compared with normal liver mitochondria [22], this study is showing for the first time that a comparable phenomenon also occurs in liver mitochondria isolated from rats bearing a chemically induced tumor located far from the liver.

As expected, the alterations in mitochondrial plasticity affected ionic exchanges. Potassium concentration was specifically decreased in the liver mitochondria of cancerous rats exhibiting the most pronounced acceleration of H_2_Ost dynamics (T+++), which appeared to represent a late event. This suggests an unbalance between the potassium efflux mediated by the K^+^/H^+ ^antiporter and the influx mediated by the mitochondrial K_ATP _channel [23]. It has been mentioned that mitochondrial K_ATP _is involved in protecting cells from ischemia and reperfusion injury, particularly in altering the rate of mitochondrial ROS production [24]. The functional properties of the main channel type for mitochondria potassium-selective transport, K_ATP_, could be affected during the final step of tumor development. Simultaneously, an increase in the Cu^2+ ^content of liver mitochondria was observed. Early reports have reported modifications of copper metabolism in malignant-tumor-bearing mice, characterized by an increased copper concentration in erythrocytes and plasma [25]. The liver has a central role in whole-body copper metabolism. The levels of copper are higher in the liver than in any other organ, and its subcellular distribution shows that about 20 per cent of hepatic copper is distributed in mitochondria plus lysosomes and peroxisomes [26]. The increase in copper and parallel decrease in manganese suggest that among cupro-proteins the cytochrome *c *oxydase [27] would be more likely involved than the mitochondrial superoxide dismutase in the observed elevation of mitochondrial copper content of brain tumor-bearing rats.

Liver mitochondria of tumor-bearing rats exhibited a decrease in mitochondrial manganese concentration. Early findings based on NMR analysis measurements of tumor-bearing mice have pointed to significant longer proton relaxation times of tumor tissues when compared with their normal counterpart [28,29]. It was later demonstrated that this effect could be caused by a decrease in the content of paramagnetic ions (Mn^2+^, Cu^2+ ^and Fe^2+^), of which manganese was the most pronounced [30]. However, the concentrations of Mn^2+ ^are so small that even the changes in this ionic component could not possibly be responsible for the changes observed in the relaxation times between liver mitochondria from healthy and brain tumor-bearing rats. The cholesterol-induced perturbation in mitochondrial fluidity could also contribute to modify ion concentrations as the voltage dependent anion channel (VDAC) located in the outer membrane is a key component of the permeability transition pore.

Compared with our recent study of apoptotic mitochondria generated in the liver of rats by injection of chemicals, the evolution of water dynamics in liver mitochondria from brain tumor-bearing rats presents a number of analogies and some slight differences. As in apoptotic mitochondria (HAM) [7], the parameters of structured water in the brain tumor-bearing rats (decrease in ***τ***_*D*_, increase in *E*_*D*_) and of total water (rise in *T1obs *and *T2obs *values) evolved in the same way, compared with those of healthy rats. The greater amplitude of variation in ***τ***_*D*_, and *E*_*D *_especially observed in the T+++ subgroup could be explained by changes produced in the mitochondrial lipid composition of the membranes [31] and in mitochondrial ion concentrations which are likely to induce alterations of hydration shells and transconformation of the macromolecules [32]. In addition, the increase in the cholesterol content of the mitochondrial membranes is likely to contribute to the discrepancy between the observed dramatic elevation of the relaxation times of structured water and the limited rise in the relaxation times of total water. The confrontation of NMR data on mitochondrial water and evolution of caspase activity in the liver tissue suggests that the significant elevation of caspases in brain tumor-bearing rats is associated to increased hepatocyte apoptosis, a phenomenon which could be related to a higher level of oxidative stress [33]. The fact that liver mitochondria enriched in cholesterol exhibits impaired function of the permeability transition pore [8] also suggests that the elevated cholesterol content observed in the liver mitochondria from the brain tumor-bearing rats contributes to the deregulation of mitochondrial biogenesis.

## Conclusion

This study enlightens the sensitivity of the structured water phase in the liver mitochondria machinery to external conditions. Our data are consistent with the idea that the acceleration of the dynamics of structured water could account, almost partly, for the profound metabolic and functional changes affecting liver mitochondria which are linked to the tumor development at a distant site, leading to abnormal features of ion transport mitochondrial biogenesis and apoptosis.

Systemic changes induced by cancer occurrence may reflect the influence of chronic inflammation on energy metabolism and the release of active molecules by the tumor, and account partly for the profounds alterations in the body composition that characterize cachexia.

## Abbreviations

SELDI-TOF: Surface-enhanced laser desorption/ionization time-of-flight, MUFA: monounsaturated fatty acids, PUFA : polyunsaturated fatty acids, GSH : glutathione, NMR : Nuclear magnetic resonance, PTP : Permeability transition pore, DEVD: Asp-Glu-Val-Asp, ENU : ethylnitrosourea, H_2_Ost: structured water, ROS : reactive oxygen species, Mn-SOD : manganese superoxide dismutase.

## Competing interests

The author(s) declare that they have no competing interests.

## Authors' contributions

DP conceived of the study, acquired and analysed the data and drafted the manuscript; CO and ED made substantial contributions to the acquisition, analysis and interpretation of the data and were involved in drafting the manuscript; KM, FMV and JM made substantial contributions to the design and interpretation of the data and revised critically the manuscript for important intellectual content.

All authors read and approved the final manuscript.

## Pre-publication history

The pre-publication history for this paper can be accessed here:



## Figures and Tables

**Table 1:** Comparison of the changes in physiological and NMR parameters of total water in liver mitochondria induced by tumor development and fasting.

	**Body weight loss**	**Liver to body weight ratio**	**T1obs**	**T2obs**
**Tumor-bearing/Healthy rats (n = 5)**				
Total (n = 22)	-22% (****)	-16% (***)	+ 11%(*)	+ 6% (NS)
T+++ (n = 3)	-23% (****)	-27% (***)	+ 36% (*)	+ 29% (*)

**Fasted/Fed rats **(n = 6/n = 6)	-8% (*)	-23% (**)	+ 26% (**)	+ 55% (****)

Statistical significance (Student's *t*-test) between groups means:

(*) 0.02 <*P *< 0.05, (**) 0.01 <*P *< 0.02, (***) 0.001 <*P *< 0.01, (****) *P *< 0.001.
